# Sensitive Detection of Gene Expression in Mycobacteria under Replicating and Non-Replicating Conditions Using Optimized Far-Red Reporters

**DOI:** 10.1371/journal.pone.0009823

**Published:** 2010-03-23

**Authors:** Paul Carroll, Lise J. Schreuder, Julian Muwanguzi-Karugaba, Siouxsie Wiles, Brian D. Robertson, Jorge Ripoll, Theresa H. Ward, Gregory J. Bancroft, Ulrich E. Schaible, Tanya Parish

**Affiliations:** 1 Barts and The London School of Medicine and Dentistry, Queen Mary University of London, London, United Kingdom; 2 Department of Medicine, Imperial College London, London, United Kingdom; 3 Department of Molecular Medicine and Pathology, University of Auckland, Auckland, New Zealand; 4 Institute of Electronic Structure and Laser, Foundation for Research and Technology-Hellas, Heraklion, Greece; 5 Department of Infectious and Tropical Diseases, London School of Hygiene and Tropical Medicine, London, United Kingdom; 6 Department of Molecular Infection Research, Research Center Borstel, Borstel, Germany; 7 Infectious Disease Research Institute, Seattle, Washington, United States of America; Institut de Pharmacologie et de Biologie Structurale, France

## Abstract

Fluorescent reporter proteins have proven useful for imaging techniques in many organisms. We constructed optimized expression systems for several fluorescent proteins from the far-red region of the spectrum and analyzed their utility in several mycobacterial species. Plasmids expressing variants of the *Discosoma* Red fluorescent protein (*Ds*Red) from the *Mycobacterium bovis hsp60* promoter were unstable; in contrast expression from the *Mycobacterium smegmatis rpsA* promoter was stable. In *Mycobacterium tuberculosis* expression of several of the far-red reporters was readily visualised by eye and three reporters (mCherry, tdTomato, and Turbo-635) fluoresced at a high intensity. Strains expressing mCherry showed no fitness defects *in vitro* or in macrophages. Treatment of cells with antibiotics demonstrated that mCherry could also be used as a reporter for cell death, since fluorescence decreased in the presence of a bactericidal compound, but remained stable in the presence of a bacteriostatic compound. mCherry was functional under hypoxic conditions; using mCherry we demonstrated that the P_mtbB_ is expressed early in hypoxia and progressively down-regulated. mCherry and other far-red fluorescent proteins will have multiple uses in investigating the biology of mycobacteria, particularly under non-replicating, or low cell density conditions, as well as providing a novel means of detecting cell death rapidly.

## Introduction

The mycobacteria include a number of pathogens of global importance such as *Mycobacterium tuberculosis*, *Mycobacterium leprae*, *Mycobacterium bovis* and *Mycobacterium avium*. *M. tuberculosis*, the causative agent of tuberculosis in humans, is one of the leading causes of mortality in the world accounting for approximately two million deaths per year. The bovine pathogen, *M. bovis*, also causes disease in humans but has a greater economic impact on farming and trade. The *M. tuberculosis* complex species, which include *M. tuberculosis* and *M. bovis*, are slow-growing organisms which require specialized biosafety facilities, possess a characteristic lipid-rich cell wall and a GC-rich genome, all of which have made studies difficult and time-consuming. Much research has focused on the generation of novel molecular techniques to aid in the study and combat of the pathogenic mycobacteria, especially *M. tuberculosis*.

Since the 1940s when antibodies were tagged with fluorescein isocyanate to aid detection in histopathological sections [1], fluorescence has been a valuable tool in biological research. Fluorescent reporters have been used to investigate transcriptional activity, for protein localisation, to screen microbial population dynamics *in situ*, and for comparative genomic, transcriptomic, and proteomic studies. Green fluorescent protein (GFP) was first isolated in 1960 from *Aequorea victoria*, but it was not until 1994 that GFP was first utilised to report gene expression in prokaryotes and eukaryotes [2]. GFP has excitation and emission maxima of 395nm and 509nm respectively, and has been extensively used in mycobacteria for quantifying bacilli and plasmid numbers [3], localisation of bacteria in the granuloma and macrophage [4], [5] and measuring promoter activity [6], [7], [8], [9]. Another commonly used fluorescent reporter, with homology to GFP, is *Ds*Red, originally cloned from the coral *Discosoma striata* [10]. One of the main benefits of using *Ds*Red rather than GFP is its red-shifted excitation and emission maxima (558nm and 583nm respectively). This allows for long-term excitation, since live cells are less sensitive to these wavelengths, as well as a reduction in auto-fluorescence and scattering by extending the range of resonance energy transfer [11]. However, *Ds*Red excitation and most of its emission spectrum also lies at one of the absorbing peaks of hemoglobin and is therefore not an ideal candidate for *in-vivo* imaging in small animals due to the lower depths that can be probed. Additionally, native *Ds*Red is not an ideal reporter due to the formation of tetramers which can cause toxic aggregates, and a slow maturation time via a green intermediate [12], [13], [14]. Mutant derivatives of *Ds*Red have been generated to overcome these problems, these include mCherry [15], mKate [16], mPlum [17], tdKatushka [16], tdTomato [15] and Turbo-635 [16]. These reporters have been extensively used in living eukaryotic systems and variants of the Clonetech *Ds*Red2 have been used in several mycobacterial species [18], [19], [20], [21].

Since what happens *in vitro* is not always the same as that *in vivo* [22], a recent focus has been on developing fluorescent reporter strains for use in non-invasive techniques to study host-pathogen interactions. In cancer research, the disease can be visualised *in vivo* using both bioluminescence [23], [24], [25] and fluorescence [26], [27], [28], [29], [30] in the murine model. However, the main reason that imaging infectious diseases lag behind is due to the inherent properties of the cells under investigation; eukaryotic cells are much larger in size and volume than prokaryotic cells. In prokaryotic models, *M. bovis* BCG expressing a fluorescent reporter has been visualised in exteriorized murine livers [31], [32], [33]. Similarly, fluorescent probes have been shown to traffic cellular function in exteriorized murine kidneys and also as a marker for pathogenesis [34] and leukocyte recruitment in the mouse footpad using YFP [35]. EGFP has been used as a marker for pathogenesis of the obligate intracellular protozoa *Leishmania* to study neutrophil recruitment [36] and both GFP and *Ds*Red have been used to visualise *Pseudomonas aeruginosa* colonisation of the gastrointestinal tract in Zebra fish [37].

We investigated the utility of far-red fluorescent proteins in several mycobacterial hosts. We demonstrated that mCherry, mPlum, tdTomato and Turbo-635, are all useful fluorescent reporters, which can be easily detected when expressed from mycobacterial promoters. In *M. tuberculosis*, no adverse effects on fitness were seen. We also investigated the utility of mCherry as a reporter of cell viability and density, as well as of promoter activity in replicating and non-replicating (hypoxic environments). We discuss the impact these far-red reporters will have on the future of mycobacterial research.

## Results

We wanted to develop bright fluorescent reporters to aid in our understanding of *M. tuberculosis* host interactions *in vitro* and, ultimately, *in vivo*. A large number of fluorescent reporter proteins are available, although only GFP and *Ds*Red have been applied to mycobacteria [3], [4], [5], [6], [7], [8], [9], [14], [17], [18], [19], [20]. Factors affecting the applicability of a particular fluorescent protein in a given setting include the ability to express the protein efficiently, lack of toxicity to the cells, brightness in comparison to auto-fluorescence of the sample, photostability, insensitivity to environmental conditions and minimal crosstalk with other fluorescent proteins if dual labelling is required [2]. In addition, the correct folding and oligomerisation of the protein is critical, since obligate oligomerisation of some variants can be toxic or disruptive to the cell or organism, while tandem dimerisation can enhance brightness. In order to address some of these issues, we selected several variants for evaluation. Since one of the potential applications of fluorescent reporters is for *in vivo* imaging, we focussed on the far-red reporters, where tissue penetration would be optimal.

**Figure 1:**
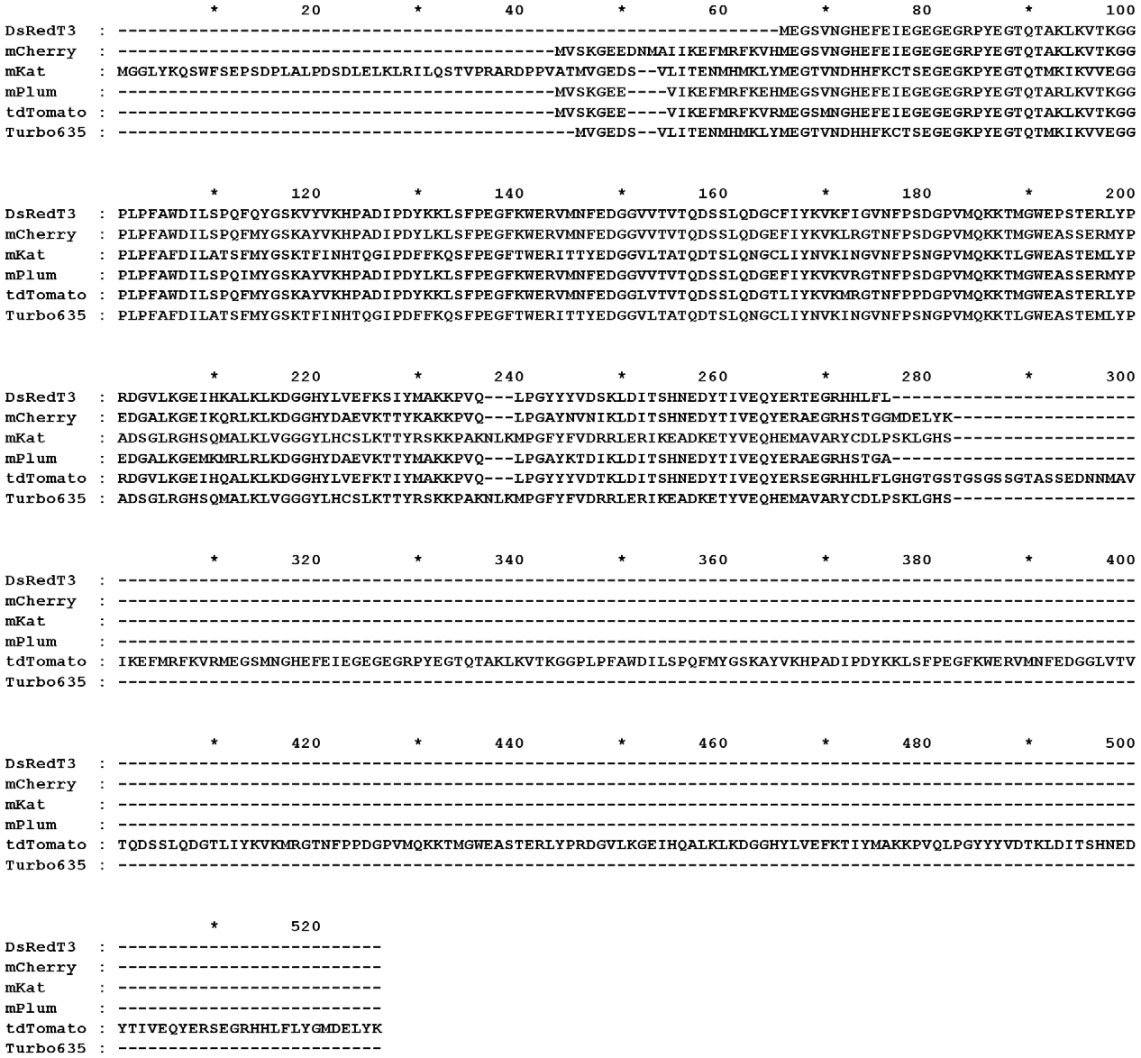
Amino-acid alignment of the far-red fluorescent proteins used in this study with DsRed. mCherry, mPlum, mKat and Turbo635 are all expressed as monomers, tdTomato is expressed as a tandem dimer. Identical residues are marked with *, similar residues are marked with : and .

We selected a range of far-red fluorescent reporters (FPs) for evaluation, namely mCherry [15], mKat, mPlum [17], tdTomato [15] and Turbo-635 [16]. All are derived from *Ds*Red and emit at higher wavelengths than background auto-fluorescence from tissue [11]; tdTomato is expressed as a tandem dimer; all other reporters are monomers (Figure 1).

### Expression of reporters using P_hsp60_ results in plasmid instability in *M. smegmatis*

Each reporter gene was optimised for *M. tuberculosis* codon usage, synthesised and cloned under the control of the strong, constitutive promoter P_hsp60_ [38]. Two expression vectors were used, differing only in their copy number; pSMT3 is a low copy number vector and we also engineered a higher copy number variant (pSMT3-M), with approximately 5–7-fold higher copy number than the wild-type plasmid by removal of an arginine residue at position 364 in the pAL5000 replicon (ΔA_364_) [39].

**Figure 2:**
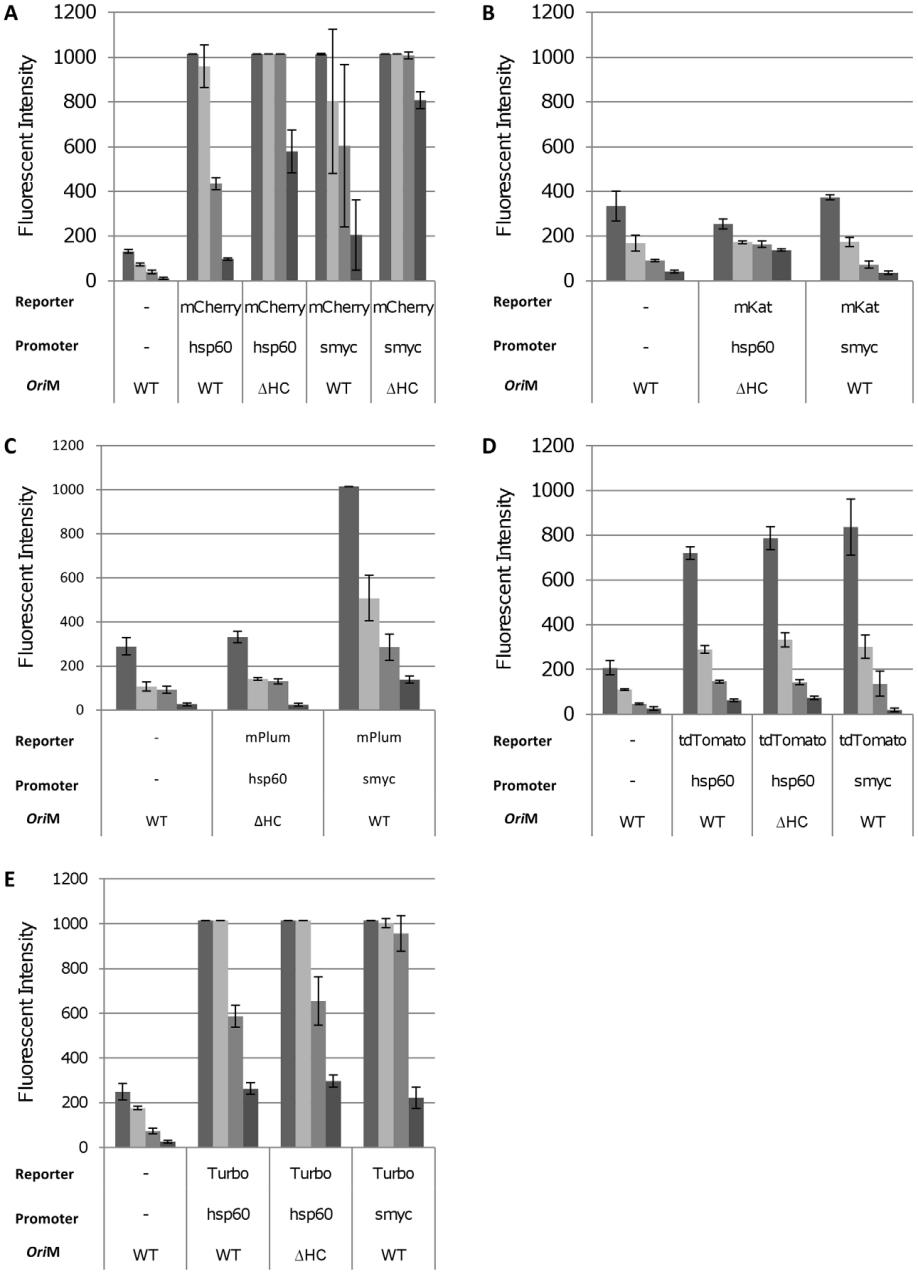
Detection of fluorescent reporters in *E. coli* DH5α. *E. coli* transformants were cultured and diluted (from left to right) to an OD_580_ of 0.25, 0.10, 0.05 and 0.01, representing cell densities of 5.5×10^6^, 1.5×10^6^, 7.7×10^5^ and 1.1.×10^5^ per mL respectively. Fluorescence was quantified at the following wavelengths: mCherry 587/610 nm, mKat 588/635 nm, mPlum 590/649 nm, tdTomato 554/581 nm, Turbo-635 588/635 nm. Data are the averages and standard deviations from three independent transformants. A value of 1015 corresponds to saturation of the machine. The reporter, promoter and vector backbone are indicated. WT = wild-type oriM; ΔHC – high copy number derivative oriM.

Since the *hsp60* promoter is functional in *E. coli* as well as in mycobacteria, constructs were tested in *E. coli* for fluorescence (Figure 2). Expression of all FPs resulted in coloured colonies; fluorescence measurements confirmed that mCherry, tdTomato and Turbo-635 were measurable, whereas mKat and mPlum were not.

**Figure 3:**
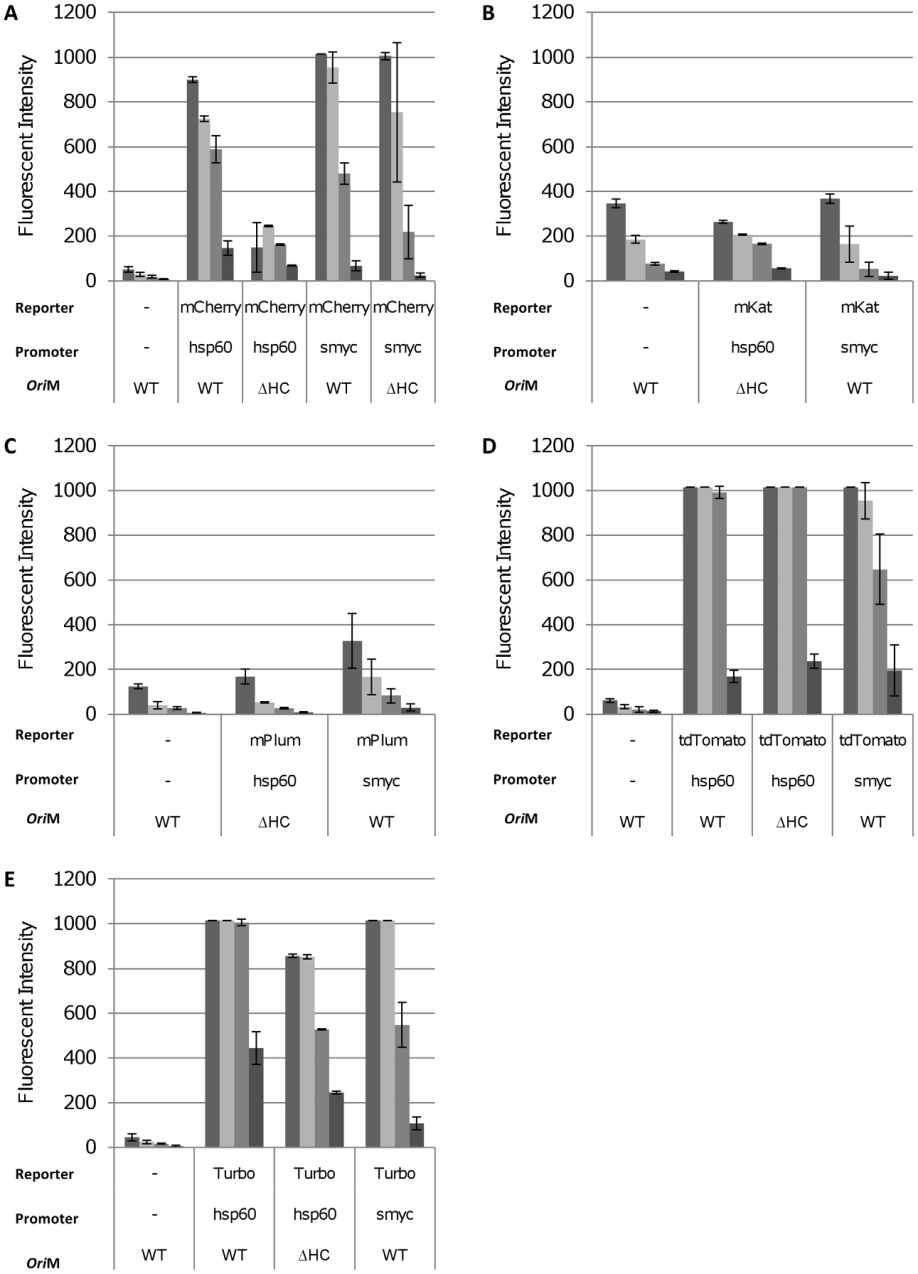
Far-red fluorescent reporters are functional in *M. smegmatis* mc^2^155. Fluorescent reporters were expressed from P_hps60_ or P_smyc_ in *M. smegmatis* and assayed in liquid culture. Cultures were diluted (from left to right) to an OD_580_ of 0.25, 0.10, 0.05 and 0.01, representing cell densities of 7.8×10^7^, 2.3×10^7^, 9.2×10^6^ and 1.8×10^6^ per mL respectively. Fluorescence was quantified at the following wavelengths: mCherry 587/610 nm, mKat 588/635 nm, mPlum 590/649 nm, tdTomato 554/581 nm, Turbo-635 588/635 nm. Data are the mean and standard deviation from three independent transformants. A value of 1015 corresponds to saturation of the machine. The reporter, promoter and vector backbone are indicated. WT = wild-type oriM; ΔHC – high copy number derivative oriM.

**Figure 4:**
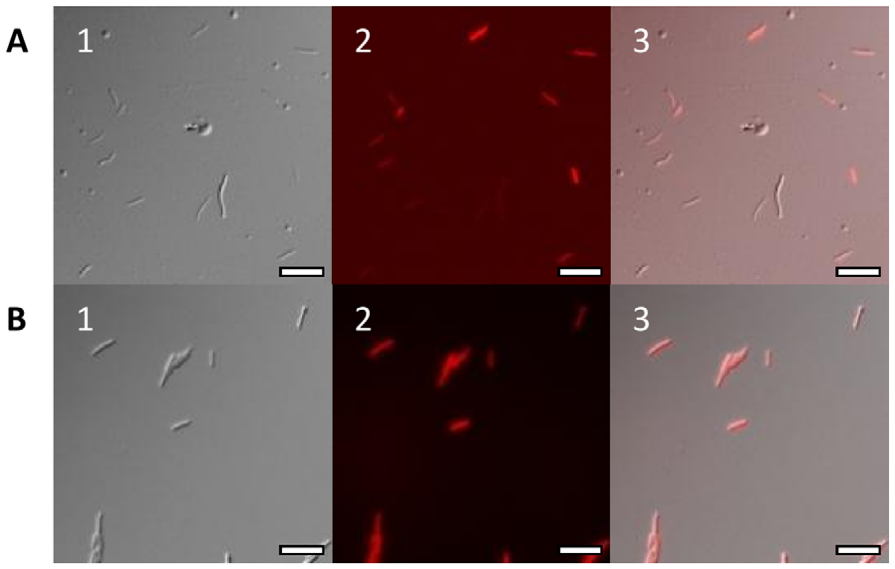
Microscopy of *M. smegmatis* mc^2^155 strains expressing mCherry. *M. smegmatis* transformants were grown in liquid for microscopy. Cells were visualised by differential interference contrast and fluorescence microscopy. Panel A - transformants carrying P_hsp60_-mCherry. Panel B - transformants carrying P_smyc_-mCherry. 1 - Differential interference contrast; 2 - Texas Red filter; 3 - overlaid image. Scale bar is equal to 10 µm.

Similarly, in *M. smegmatis*, all of the fluorescent reporters, except mKat and mPlum, gave rise to a proportion of coloured colonies indicating successful expression in a subset of cells. For each reporter, liquid cultures were prepared from coloured colonies. A fluorescence scan to determine the optimal excitation and emission wavelengths was carried out and fluorescence was measured at the optimum wavelengths for varying cell densities (Figure 3). Low background fluorescence was seen from the cells. Strong signals were seen from mCherry, tdTomato and Turbo; fluorescence was still measurable above background at cell densities as low as 1×10^6^ per mL (OD_580_ = 0.01). No signal above background was seen with either mPlum or mKat. Contrary to expectations, fluorescence from the increased copy number plasmids was not higher than for the low copy number plasmid and in fact for two reporters (mCherry and Turbo) was lower. On further inspection of these strains, two populations of cells were found - coloured and non-coloured. The increased copy number plasmid yielded fewer coloured colonies than the wild-type plasmid. This indicated that there was a stability issue originating from the ΔA_364_ mutation. Using fluorescent microscopy to visualise the fluorescent strains confirmed that only a proportion of the bacilli expressed the fluorescent reporter (Figure 4). Plasmids recovered from non-fluorescent colonies had deletions in the promoter-FP region, whereas plasmids recovered from the coloured colonies appeared intact (data not shown), confirming that loss of expression was due to the acquisition of deletions in the plasmids.

### Expression of reporters using P_smyc_ is stable in *M. smegmatis*

Since expression from P_hsp60_ was unstable, we used an alternative promoter. *M. smegmatis* P_rpsA_ is a strong promoter, which results in high-level expression [40]. Since high-level expression could give rise to selection pressure due to toxicity, we used a version of this promoter engineered to contain the TetO operator (P_smyc_), such that expression could be regulated by the addition of the TetR repressor [40]. This would give us the flexibility to down-regulate expression if it compromised strain fitness. Vectors were constructed to express all reporters from P_smyc_ in the same plasmid background as for P_hsp60_, either using the low copy number vector, or the increased copy number version (Figure 3).

As before, transformation of *E. coli* and *M. smegmatis* resulted in coloured colonies for mCherry, tdTomato and Turbo; mPlum also gave rise to coloured colonies. Expression of mCherry from P_smyc_ in liquid culture was comparable to expression from P_hsp60_ in *M. smegmatis*, whilst expression of tdTomato and Turbo was slightly lower for P_smyc_. Expression of mPlum was seen from the P_smyc_ promoter, although in comparison to the other reporters, mPlum was not as bright. mKat fluorescence could not be detected from P_smyc_. Microscopy confirmed that all of the bacilli expressed the FP driven by P_smyc_ (Figure 4).

### Fluorescent reporters are functional in *M. marinum*

Having demonstrated high-level fluorescence using far-red reporters in *E. coli* and *M. smegmatis*, we wanted to determine if the same reporters functioned in other mycobacteria. *M. marinum* is a pathogen of ectothermic organisms adapted to growth at lower temperatures (25–35°C). It has been used as a pathogenicity model for *M. tuberculosis* and fluorescent reporters have been previously used in this species [41], [42], [43]. Since *M. marinum* produces a yellow photochromogenic pigment in the presence of light, we grew recombinant strains at 32°C in the dark and in the light.

**Figure 5:**
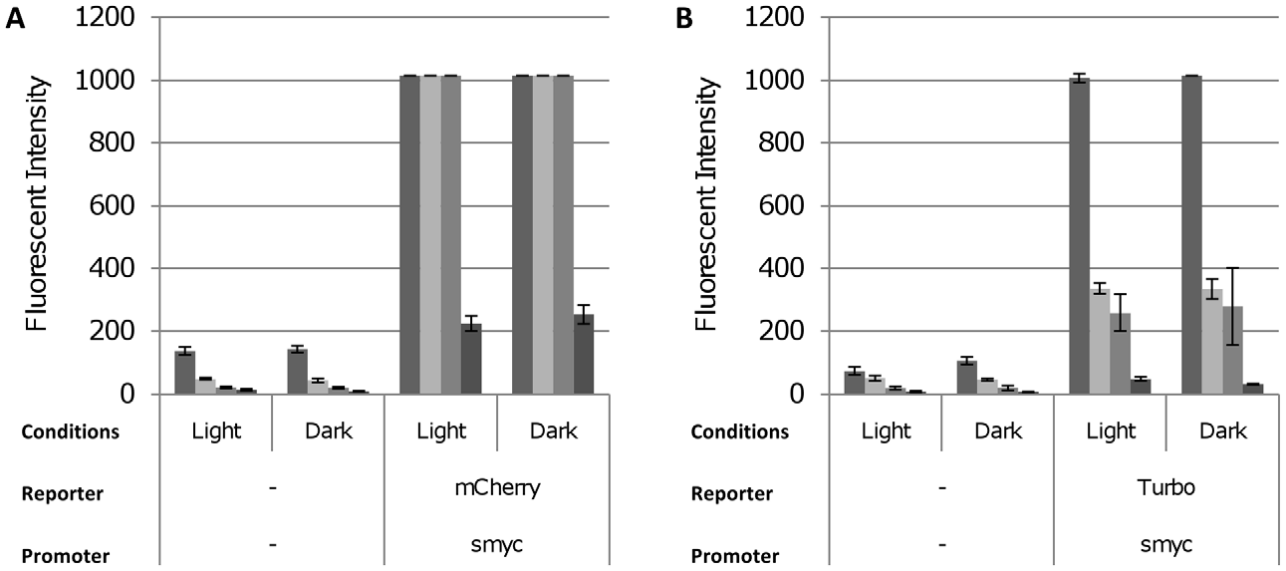
Far-red fluorescent reporters are functional in *M. marinum* strain M. Fluorescent reporters were expressed from P_smyc_ in *M. marinum* and assayed in liquid culture grown either in the dark or in the light. Cultures were diluted (from left to right) to an OD_580_ of 0.25, 0.10, 0.05 and 0.01, representing cell densities of 9.8×10^5^, 4.2×10^5^, 1.9×10^5^ and 6.5×10^4^ CFU/ml respectively. Fluorescence was quantified at the following wavelengths: mCherry 587/610 nm, Turbo-635 588/635 nm. Data are the mean and standard deviation from three independent transformants. A value of 1015 corresponds to saturation of the machine. The reporter and promoter backbone are indicated.

We tested mCherry and Turbo and found that both were highly fluorescent in *M. marinum* (Figure 5). No difference in background fluorescence was seen between cultures grown in the light or in the dark. In addition, there was no difference in the fluorescence emitted from either mCherry or Turbo in cultures grown in the dark or light. Both reporters gave sensitive detection of bacterial numbers, being able to detect a minimum of 6.4×10^4^ CFU/ml, comparable to that seen with *M. smegmatis*.

### High-level fluorescence of reporters was detected in *M. tuberculosis*

**Figure 6:**
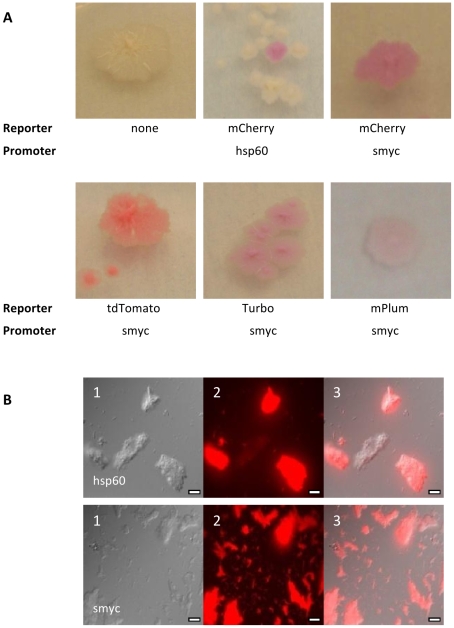
Detection of fluorescent reporter in *M. tuberculosis* H37Rv. FP expression plasmids were transformed into *M. tuberculosis* and colonies isolated on hygromycin-containing plates. (A) Expression of several fluorescent reporters gave rise to coloured colonies. (B) Microscopy of fluorescent mCherry reporter driven by either P_hsp60_ or P_smyc_. Scale bar is equal to 10 µm.

Since we had demonstrated stable, high level expression of FPs in both *M. smegmatis* and *M. tuberculosis*, we decided to test the same plasmids in *M. tuberculosis*. Reporter plasmids were transformed into *M. tuberculosis* and assessed for fluorescence. We tested expression of Turbo, tdTomato, mCherry, mKat and mPlum expressed from P_hsp60_ or P_smyc_. For mCherry we also compared the low and increased copy number plasmids. All of the fluorescent reporters expressed from P_smyc_, except mKat generated coloured colonies in *M. tuberculosis* H37Rv (Figure 6). Transformants containing tdTomato were bright pink, whereas mCherry, Turbo-635 and mPlum were varying intensities of purple. For each of these vectors, 100% of the transformants were coloured. Microscopy confirmed that all of the bacterial cells in the population were expressing mCherry from P_smyc_ (Figure 6). In contrast, only a small proportion of transformants were coloured when the reporter was expressed from P_hsp60_; less than 1% of the transformants for tdTomato, Turbo and 5% for mCherry. Again, this suggests that stability is an issue using the *hsp60* promoter for expression. In addition, the use of the increased copy number vector caused a further reduction in the number of coloured transformants. For example, using mCherry, 5% of the transformants were coloured with P_hsp60_, 1% with P_hsp60_ in pSMT3-M, 100% with P_smyc_, and 26% with P_smyc_ in pSMT-3M.

**Figure 7:**
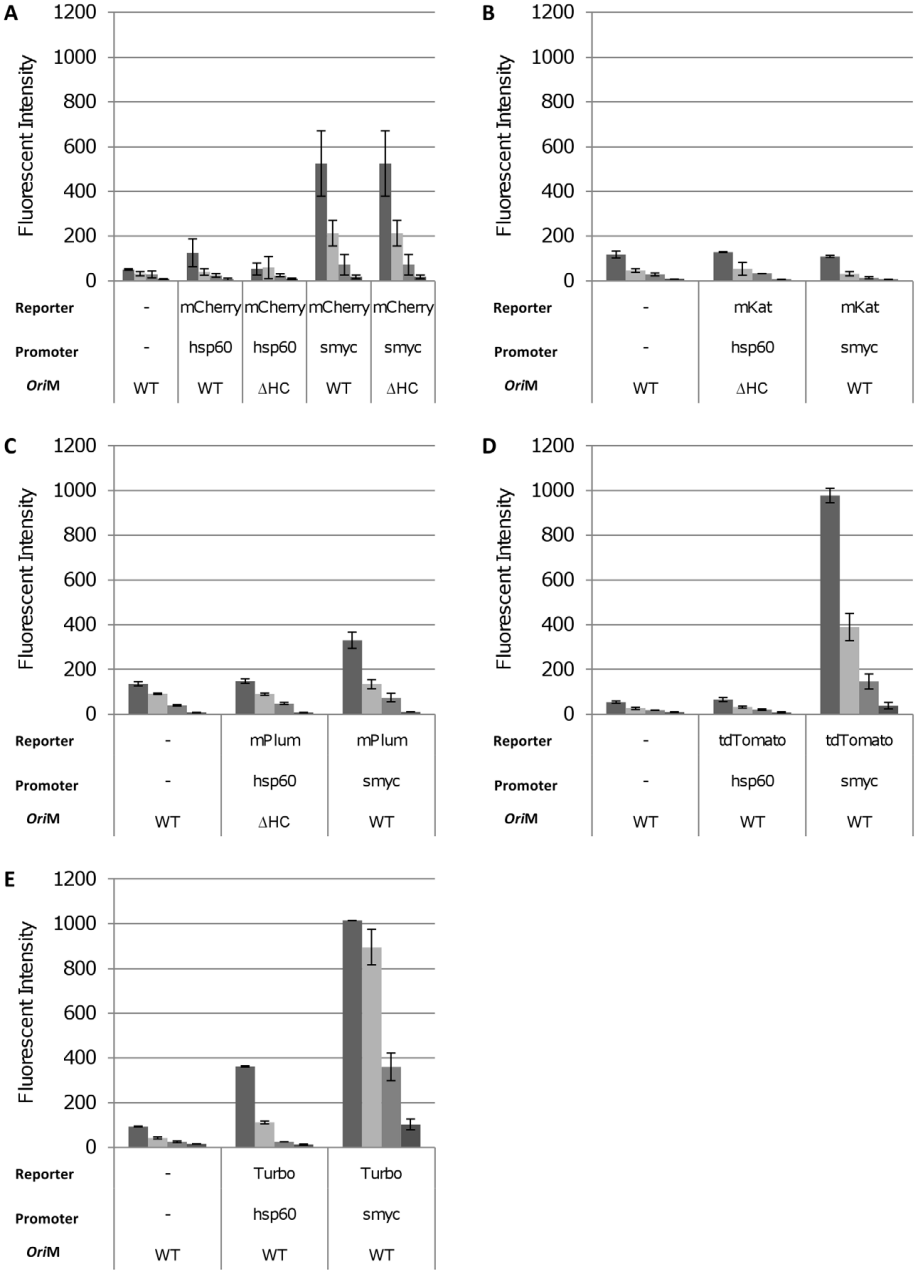
Detection of fluorescent reporters in liquid cultures of *M. tuberculosis* H37Rv. Fluorescent reporters were expressed from P_hps60_ or P_smyc_ in *M. tuberculosis* and assayed in liquid culture. Cultures were diluted (from left to right) to an OD_580_ of 0.25, 0.10, 0.05 and 0.01, representing cell densities of 4.8×10^7^, 1.7×10^7^, 9.1×10^6^ and 1.7×10^6^ CFU/ml respectively. Fluorescence was quantified at the following wavelengths: mCherry 587/610 nm, mKat 588/635 nm, mPlum 590/649 nm, tdTomato 554/581 nm, Turbo-635 588/635 nm. Data are the mean and standard deviation from three independent transformants. A value of 1015 corresponds to saturation of the machine. The reporter, promoter and vector backbone are indicated. WT = wild-type oriM; ΔHC – high copy number derivative oriM.

Fluorescence was measured in *M. tuberculosis* in liquid culture (Figure 7). For all of the reporters tested, P_smyc_ gave the highest fluorescent signal. Expression levels from P_hsp60_ were much lower for all reporters, and in fact a signal over background could only be seen for Turbo and mCherry at high cell densities (OD_580_ = 0.25, 4.8×10^7^ CFU/ml). Microscopy indicated that all the bacilli in which FP expression was driven by P_smyc_ were fluorescent (Figure 6). In contrast, only a fraction of the bacilli expressed FPs under the control of P_hsp60_ (Figure 6). This is in agreement with the data generated in *M. smegmatis*, demonstrating that plasmid instability was mediated by P_hsp60_.

### P_smyc_-reporter plasmids are stable in *M. smegmatis* and *M. tuberculosis*

High-level expression of heterologous genes can be detrimental to bacterial fitness and has been posed to be the reason for plasmid instability [44]. In addition, the expression of fluorescent proteins themselves has been linked to loss of virulence or stress tolerance [44]. Since we had seen plasmid instability using P_hsp60_ for expression, but not P_smyc_ expression, we reasoned it was unlikely that expression of the fluorescent reporters *per se* was the selection pressure driving plasmid rearrangements. In order to investigate this, we looked at plasmid stability and bacterial strain fitness during a number of growth conditions.

**Figure 8:**
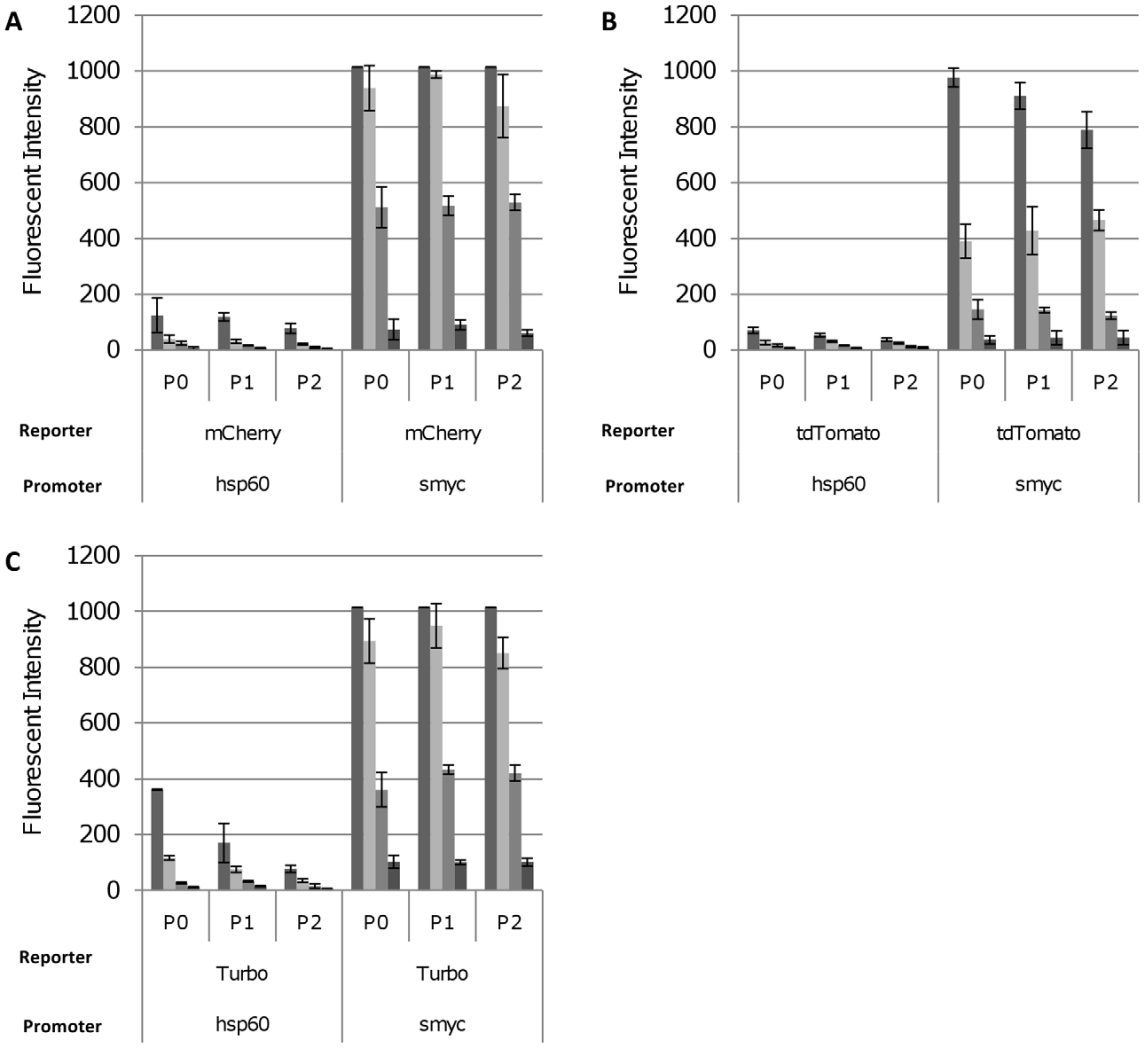
Expression of fluorescent proteins in *M. tuberculosis* H37Rv is stably maintained. Transformants were grown in liquid medium and passaged every 5 days into fresh medium. Fluorescence was quantified at wavelengths associated with optimum reporter expression and emission at cultures normalised to OD_580_ of 0.25, 0.10, 0.05 and 0.01 (from left to right). Fluorescence was quantified at the following wavelengths: mCherry 587/610 nm, tdTomato 554/581 nm, Turbo-635 588/635 nm. Data are the averages and standard deviations from three independent transformants. A value of 1015 corresponds to saturation of the machine. The reporter, promoter and vector backbone are indicated. P0 = initial culture; P1 = passage 1; P2 = passage 2.

*M. smegmatis* and *M. tuberculosis* transformants carrying the three brightest reporters (mCherry, tdTomato and Turbo) expressed from P_hsp60_ or P_smyc_ in the low copy number vector were cultured in liquid medium and serially passaged. Fluorescence was measured in liquid cultures (Figure 8) over three passages. In *M. tuberculosis*, there was no diminution of the fluorescence signal in P_smyc_-reporter plasmids, all of which retained high signals. In contrast, for P_hsp60_-driven expression, in the only vector in which expression could be detected (Turbo), expression decreased over time, suggesting accumulation of deletions or mutations in the plasmid. Fluorescence intensity from the *M. smegmatis* strains followed a similar trend (data not shown). These data confirmed that expression from P_hsp60_ was unstable and that expression decreased over time. This appears to be an inherent property of P_hsp60_, since it was not related to the level of expression of fluorescence and the use of P_smyc_ allowed stable long-term expression.

### High-level expression of reporters does not compromise growth in *M. tuberculosis*

**Figure 9:**
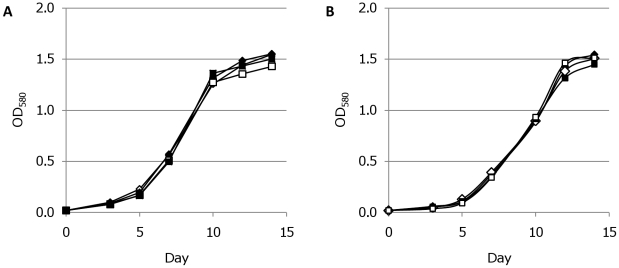
Growth kinetics of *M. tuberculosis* reporter strains. *M. tuberculosis* transformants were grown in liquid medium in the (A) presence or (B) absence of hygromycin. ◊ pSMT3 empty vector control; □ tdTomato expressed from P_smyc_; Δ Turbo expressed from P_smyc_; ▪ mCherry expressed from P_hsp60_ and ♦ mCherry expressed from P_smyc_. Data are the averages from three independent transformants.

**Figure 10:**
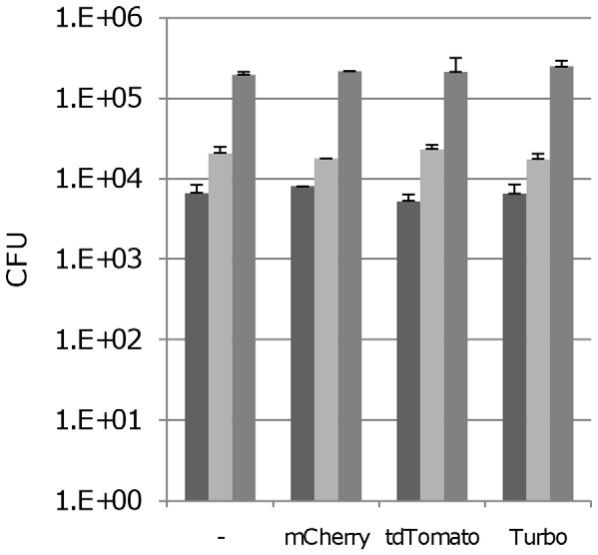
Virulence of *M. tuberculosis* fluorescent strains in murine macrophages. Murine macrophages were infected at an MOI of 1∶1 with recombinant strains expressing the indicated reporter from P_smyc_. Bacterial counts were measured over a period of 7 days. Data are the mean and standard deviation from three independent infections. Time points for each reporter strain, from left to right are day 1, day 3 and day 7.

We examined the fitness of strains expressing high levels of FPs. Growth in liquid medium was unaffected (Figure 9), confirming that there was no detrimental effect on fitness. Expression of FPs has been linked to a loss of virulence [44], so we looked at the ability of strains to replicate and survive inside macrophages (Figure 10). Strains expressing mCherry, tdTomato or Turbo were compared to wild-type in resting J774 murine monocytes. All strains showed a similar ability to survive and replace over the time of the assay (up to 7 days), increasing in numbers over time. Thus expression of FPs did not affect virulence of the strains in this assay. All colonies derived from the macrophage-passaged bacteria were coloured, confirming that the plasmid and FP expression was stably maintained over 7 days in the absence of antibiotic selection.

### Fluorescent reporters as markers of cell viability

**Figure 11:**
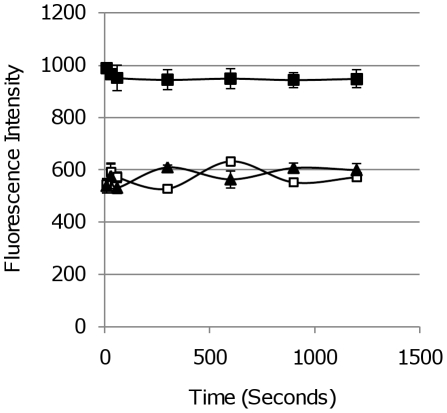
Stability of *M. tuberculosis* fluorescent strains after long-term excitation. *M. tuberculosis* transformants expressing FPs from P_smyc_ were grown in liquid medium and the OD_580_ adjusted to 0.01. Fluorescence was quantified after excitation over a period of 20 min. (▴) mCherry, (▪)Turbo-635 and (□) tdTomato.

Fluorescence can be susceptible to photobleaching after repeated or extended periods of exposure to the excitation wavelength. To determine if this might be a problem when serially exciting FPs we looked at fluorescence after long exposure times to the excitation wavelength. *M. tuberculosis* strains expressing mCherry, tdTomato and Turbo were exposed to excitation at their optimal excitation wavelength for over 2,000 seconds. No decrease in fluorescence was displayed by any of the reporter tested after long-term excitation (Figure 11).

Once we had determined that repeated measurement of FPs would not lead to a decrease in the signal from photo-bleaching, we looked at the stability of the protein in live and killed cells. We were interested to see if fluorescence could be correlated with cell viability. This would allow us to use it as a rapid readout of bacterial numbers as an alternative to the lengthy colony counts (taking 3–4 weeks). To assess this we compared the effect of bacterostatic and bactericidal compounds on fluorescence.

**Figure 12:**
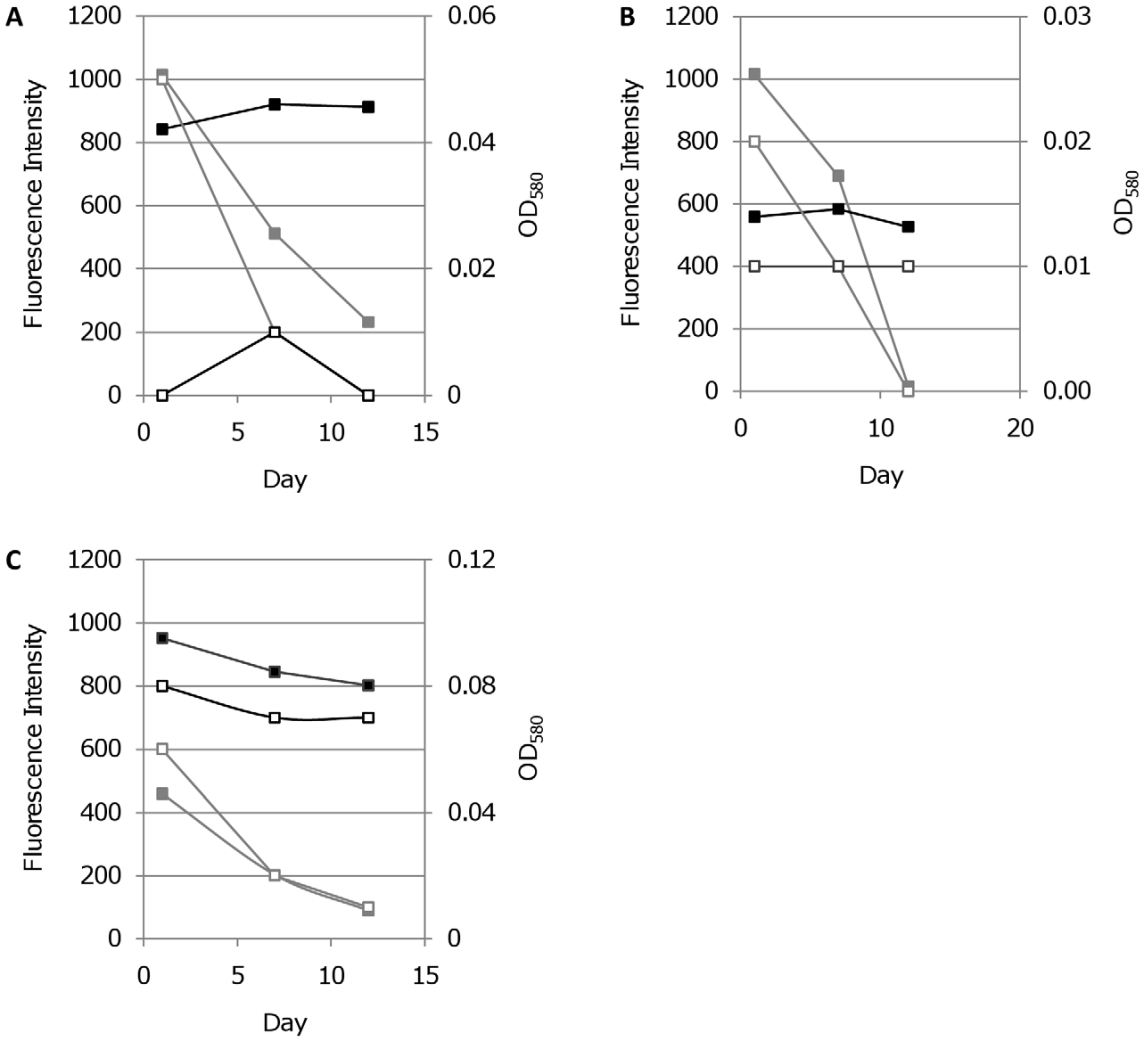
Correlation between cell viability and fluorescence in *M. tuberculosis* during antibiotic treatment. Transformants expressing FPs from P_smyc_ were grown in liquid and treated with the bactericidal antibiotic kanamycin (grey line) or the static antibiotic chloramphenicol (black line). Fluorescence intensity (solid squares) and OD_580_ (open squares) were measured. A. mCherry, B. tdTomato and C. Turbo-635.

Chloramphenicol inhibits protein synthesis in bacteria via inhibition of peptidyl transferase and has a static effect on bacterial cultures [45]. We treated *M. tuberculosis* expressing mCherry, tdTomato, or Turbo, with chloramphenicol and measured fluorescence over time. Chloramphenicol exerted a bacteriostatic effect, preventing further bacterial replication, as evidenced by the lack of increase in OD_580_ (Figure 12). Fluorescent signals mirrored the OD_580_, and no decrease in intensity was seen over 12 days. In contrast treatment with kanamycin, a bactericidal aminoglycoside, resulted in a rapid decrease in both OD_580_ and fluorescence. These data demonstrate that in dead cells, the FPs are degraded over time and are not stable enough to persist, whereas in live cells, FPs are stable over time, even in the absence of *de novo* protein synthesis. Thus, live cells could rapidly be quantitated and distinguished from dead cells in a population without the need to determine CFUs on agar plates.

### Effects of hypoxia on fluorescence in *M. smegmatis*

One of the limitations of FPs is that they require molecular oxygen for maturation of the fluorophore and therefore may not function under anaerobic conditions. *M. tuberculosis* is found in granulomas during infection, which are thought to be predominantly hypoxic, thus it would be useful to have a FP that functioned under these conditions. We evaluated the functionality of our FPs using an *in vitro* mode of hypoxia – the Wayne model. *M. smegmatis strains* expressing FPs were cultured in sealed tubes in conditions designed to generate aerobic or hypoxic conditions. For aerobic cultures, the head space ratio was 1.0 (v/v) and for hypoxic cultures it was 0.5 (v/v). Methylene blue was used as a reporter of oxygen depletion. Growth and fluorescence were measured over time.

**Figure 13:**
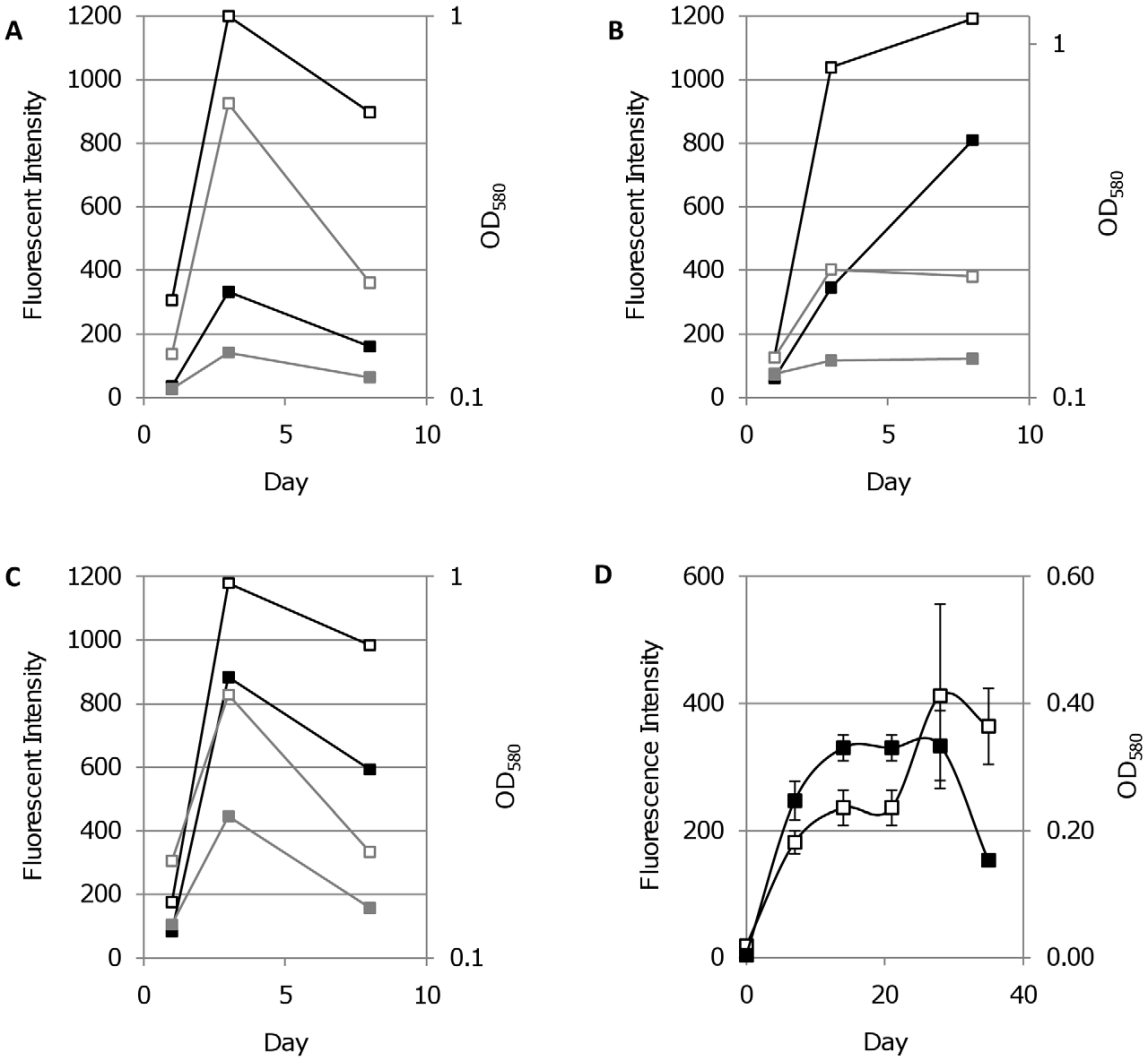
FP activity under hypoxic conditions. *M. smegmatis* (A to C) transformants expressing FPs from P_smyc_ were cultured aerobically (black lines) or under limiting oxygen (grey lines) conditions. Fluorescence intensity (solid squares) and OD_580_ (open squares) were measured. A - *M. smegmatis* expressing mCherry. B -*M. smegmatis* expressing tdTomato. C - *M. smegmatis* expressing Turbo-635. D - *M. tuberculosis* expressing mCherry under hypoxic conditions. *M. tuberculosis* transformants expressing FPs from P_smyc_ were cultured under limiting oxygen conditions. Fluorescence intensity (open squares) and OD_580_ (solid squares) were measured.

As expected, limited growth was seen in all *M. smegmatis* cultures strains with reduced oxygen over the eight-day time-course (final OD_580_<0.6) as compared to aerobic growth (final OD_580_>1.0). Methylene blue fading and decolourisation occurred at day 3 and day 6 indicating depletion and exhaustion of oxygen respectively. Fluorescence was measured over time in both culture conditions; fluorescence intensity increased in proportion to the OD_580_ of the cultures, so that relative intensity per unit OD_580_ was the same for both aerobic and hypoxic cultures. No decrease in fluorescent signal was seen in the hypoxic cultures when compared to aerobic cultures and the signal continued to increase even after oxygen depletion occurred at day 3, confirming that continued maturation of fluorophores was occuring (Figure 13). To determine if immature FPs were present, we also measured fluorescence after the introduction of oxygen into the anaerobic tubes; no increase in fluorescence was seen, confirming that there was not a significant population of inactive fluorophores present (data not shown).

A similar experiment was conducted with *M. tuberculosis* expressing mCherry grown under hypoxic conditions (Figure 13). An increase in fluorescence during growth and shift into the non-replicating state was observed. Fluorescence increased even after the optical density stopped increasing, confirming that it was not being affected by oxygen depletion in the medium. This demonstrated that the far-red FPs could be used in hypoxia models as markers of gene expression, even during oxygen depletion.

### mCherry as a sensitive reporter of promoter activity during hypoxia in *M. tuberculosis*

Our data using *M. smegmatis* showed that FPs were functional during hypoxic culture, thus affording the possibility that they could be used as sensitive reporters of promoter activity in this model. In order to investigate this possibility we compared the use of LacZ and mCherry as reporters for *mbtB* promoter activity. Previous work has suggested that mycobactin genes might be up-regulated early in hypoxia [46], [47]. MbtB is part of the mycobactin biosynthesis cluster, producing a siderophore. The upstream region of *mbtB* was cloned upstream of LacZ or mCherry and transformed into *M. tuberculosis*. LacZ activity was measured in cell-free extracts and normalised to total protein; mCherry fluorescence was measured in whole cells and normalised to OD_580_.

**Figure 14:**
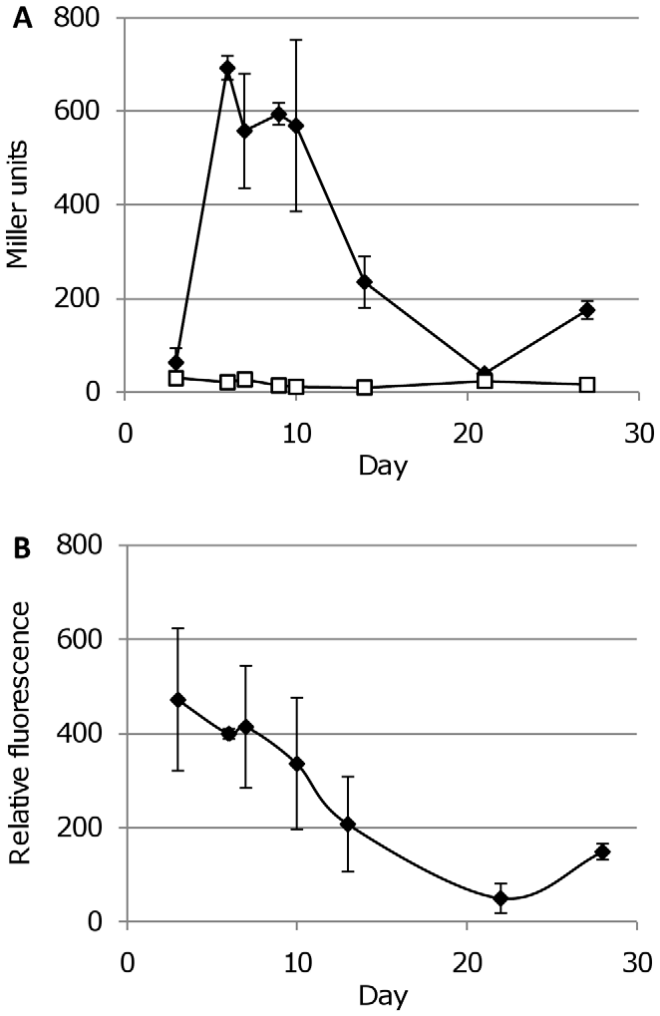
*M. tuberculosis* P_mbtB_ activity during hypoxic culture. Promoter activity was assayed in the hypoxia model using (A) LacZ and (B) mCherry as a reporter. Activity of LacZ is reported in Miller units (units activity per mg total protein in cell-free extracts). Activity from mCherry is reported in relative fluorescence units (fluorescence after background subtraction per OD_580_ in whole cells). (A) ▪ P_mbtB_ activity; □ control vector pSM128 activity. (B) ♦ P_mbtB_ activity.

LacZ expression was not detected at the earliest time points (Day 3), but was readily detected at Day 7, followed by a decrease in activity after Day 10 (Figure 14). These data suggested there could be transient induction of promoter activity occurring in response to hypoxia. However, the number of cells and therefore the protein concentrations at the earliest time point was very low, which could lead to a false negative result. This was confirmed when looking at mCherry expression, where fluorescence could be detected in the Day 3 cultures (OD_580_ = 0.08). Once fluorescent values were normalised for the number of cells (using OD_580_), the expression of mCherry decreased steadily from a high initial level (at Day 3) over the time course, indicating that there was no transient up-regulation, but that it was probably an artefact of our inability to measure LacZ activity at the earliest timepoint. Our comparison confirmed that mCherry is a more sensitive reporter than LacZ and is more suitable to conditions in which low cell densities are used. In addition, the method was non-invasive and could be performed serially on the same cultures without the need for substrate addition, simplifying measurements over time courses.

## Discussion

We have demonstrated that far-red fluorescent reporters are functional in three mycobacterial species. Fluorescence from mCherry, mPlum, tdTomato and Turbo-635 was detected at their optimum wavelengths in *M. smegmatis*, *M. marinum* and *M. tuberculosis*. The expression of these FPs from P_smyc_ resulted in colonies that were naturally coloured and did not require a substrate or specific equipment to be visualised. Of the reporters, mPlum was the dimmest, but has the advantage of the longest Em/Ex wavelengths, so that it could be used in conjunction with other far-red reporters and could be of use for visualisation in deeper tissues enabling the use of optical tomography approaches. mCherry, tdTomato and Turbo-635 all gave high levels of fluorescence allowing detection of approximately 10^6^ CFU/ml in liquid culture using a spectrofluorimeter, and providing greater sensitivity in detecting growth than turbidity.

mCherry, tdTomato and Turbo-635 were all very bright reporters and showed no photobleaching, being stable during continuous excitation. The optimal combination of FP, promoter and plasmid backbone resulted in stable, high-level expression of mCherry, tdTomato and Turbo-635. Although we incorporated the ability to regulate expression of the reporters, we found that this was not necessary. Plasmids expressing FPs from P_smyc_ gave high-level expression in *M. smegmatis*, *M. marinum* and *M tuberculosis*. Expression of mCherry from P_rpsA_ without the *TetO* operator gave the same high levels of fluorescence *in vitro* (data not shown), confirming that the operator does not affect promoter strength in the absence of the TetR repressor.

Promoter-induced vector instability has been described previously in *M. tuberculosis* [48], [49], [50], [51]. P_hsp60_ has been linked to frameshift mutations [51] and large deletions in either the promoter or coding region in *M. tuberculosis* at a frequency of 2×10^−3^, although it was reported to be stable in *M. smegmatis* [48], [49]. However, plasmid stability appears to be dependent on the specific heterologous gene being expressed [48], [52]. We confirmed that P_hsp60_ induced stability problems leading to a significant proportion of the population having deletions in the promoter-reporter loci, making this promoter unsuitable for use in these conditions.

The deletion of an alanine residue in the C-terminal of the RepA replication protein (ΔA_364_) increases the copy number of pAL5000-derived vectors approximately seven-fold [39]. Increasing the plasmid copy number had a detrimental effect on fluorescence from some of the strains leading to fewer transformants expressing the FPs. This effect was not promoter-associated, since it occurred with both P_hsp60_ and P_smyc_. We hypothesise that the increased plasmid instability results from negative selection against increased expression levels of FPs and a strong selective pressure for deletion plasmids. Bacilli with mutations in the promoter region or the reporter gene which reduce or abolish expression could have a competitive growth advantage. Alternatively, it is possible that the mutation in the plasmid affects correct partitioning and increases instability.

We demonstrated that fluorescence was detectable under hypoxic conditions *in vitro* in non-replicating cells, presumably since sufficient molecular oxygen is present in the bacterial cells to allow for fluorophore maturation. Previously, it was assumed that FPs would not function under these conditions. The ability to use optimized FPs as sensitive markers of gene expression in these conditions is of great utility since this is a non-invasive method i.e. fluorescence can be measured in whole cells under anaerobic condition without the need for addition of substrate or oxygen. Use of FPs also has the advantage of increased sensitivity at low cell densities, where other reporters may be below the limit of detection. Expression studies often use reporters of promoter activity, such as LacZ [3]. We have shown that LacZ assays using a colorimetric assay cannot be used in situations where there are low cell densities. In contrast, use of mCherry as a reporter allowed the sensitive detection of promoter activity in low cell densities without the need for cell disruption. Fluorescent reporters offer the advantages of increased sensitivity as well as the ability to measure activity sequentially in live cells. It is therefore plausible that these reporters will allow detection of bacterial gene expression profiles during long-term persistence.

Many expression studies have focussed on identifying those genes up-regulated under conditions relevant to infection. Several studies using microarrays have profiled the transcriptome in hypoxia or low oxygen conditions [46]. Under these conditions there is very little mRNA being produced since the cells transition into a non-replicating state [53], [54], [55]. Using mCherry as a reporter we showed that P_mbtB_ expression progressively decreases during hypoxia, in contrast to LacZ reporter which suggested transient up-regulation. After the earliest time points, both reporters showed that *mtbB* expression was shut off during hypoxic culture.

Fluorescence was not diminished when protein synthesis was inhibited by chloramphenicol, indicating that the FPs are stable proteins, which are not subject to high turnover rates by endogenous proteases. In addition, we were able to correlate loss of fluorescent signal with bacterial viability after exposure to a bactericidal agent. *M. tuberculosis* is a slow growing bacillus which has to be contained and manipulated in specialised facilities meaning that working on this pathogen is time consuming. The standard methodology for quantifying bacterial numbers is to determine CFUs which takes four weeks. Growth can be measured by OD, but this cannot distinguish between live and dead bacteria if cell lysis does not occur. The FP reporter strains could be used as sensitive and rapid readouts in growth studies and to identify novel anti-tubercular agents with bactericidal activity.

We demonstrated that fluorescence signal from the reporters was very stable, even after long-term excitation, allowing for serial imaging. Fitness assays demonstrated that high-level expression of FPs was not detrimental to growth in any species. Expression of these reporters did not cause any detrimental effect on the growth or virulence of *M. tuberculosis in vitro*. This suggests that optimized FPs would be suitable for *in vivo* imaging during infection studies, where repeated imaging of a virulent strain would be required. Therefore these far-red reporters may allow us to develop non-invasive tools to analyse the pathogenic events of *M. tuberculosis* in real-time and pharmacological monitoring.

We have optimized the expression of several far-red fluorescent reporters in *M. tuberculosis* by combining codon-biased synthetic genes with optimal promoters and replicons to produce reporter strains that can be detected with great sensitivity *in vitro*. mCherry was functional during hypoxia and we demonstrated that the promoter for the mycobactin operon was progressively down-regulated during hypoxia. Drug treatments confirmed that only viable cells retained the fluorescent signal, and that the signal was detectable during drug- or hypoxia-induced stasis. This should pave the way for *in vivo* studies, where sensitive detection of live bacilli in tissues is required, as well as provide novel reporters for *in vitro* studies.

## Materials and Methods

### Bacterial strains and culture conditions

*E. coli* DH5α was cultured in LB medium. *M. smegmatis* mc^2^-155 was grown in Lemco medium (10 g L^−1^ peptone, 5 g L^−1^ Lemco powder, 5 g L^−1^ NaCl) containing 0.05% w/v Tween 80 for liquid cultures or 15 g L^−1^ agar for agar plates, minimal medium as described previously [50] or Dubos-Tween Albumin (DTA) medium (Dubos medium, 0.05% w/v Tween 80, 10% v/v Dubos Albumin supplement (Becton Dickinson)). *M. tuberculosis* H37Rv (ATCC 25618) and *M. marinum* (ATCC BAA-535 /strain M) strains were grown in Middlebrook 7H9 medium plus 10% v/v OADC (oleic acid, albumin, dextrose, catalase) supplement (Becton Dickinson) and 0.05% w/v Tween 80 or on Middlebrook 7H10 agar (Becton Dickinson) plus 10% v/v OADC. Chloramphenicol was used at 8 µg/ml, hygromycin at 100 µg/ml and kanamycin at 20 µg/ml where required.

### Plasmid construction

An increased copy number version of the plasmid pSMT3 [38] was constructed using site-directed mutagenesis with the primers PAL-M1 (GGC CAT CTC GCG GAA GGG AGC GCG CAC GGC GGC) and PAL-M2 (CCG GCC GTG CGC GCT GCT CCC TTC CGC GAG ATG CCG), to generate pSMT3-M. The strong promoter P_smyc_ (*M. smegmatis rpsA* promoter with *tetO* operator) was amplified from pSE100 [40] using P_smyc_-F (TCT AGA GGA TCG TCG GCA CCG TCA C) and P_smyc_-R (GGA TCC GGA TCG TGC TCA TTT CGC G) and cloned as *Bam*HI/*Xba*I fragment into pSMT3 and pSMT3M (replacing P_hsp60_) to generate pSMT3-S and pSMT3-SM. mCherry, mKat, mPlum, tdTomato and Turbo-363 sequences were codon-optimised for *M. tuberculosis* codon preferences, synthesised (GenScript USA Inc.) and cloned into pSMT3, pSMT3-M, pSMT3-S and pSMT3-SM as *Bam*HI/*Hind*III fragments. A complete list of plasmids used in this study is in Table 1.

**Table 1:** Plasmids used in this study.

Plasmid ID	Plasmid details	Origin
pSMT3	*E. coli* - mycobacterium shuttle vector, Hyg^R^, P_hsp60_	[38]
pSMT3-M	Increased copy number pSMT3 (oriM ΔA_364_)	This study
pSMT3-S	*E. coli*-mycobacterium shuttle vector, Hyg^R^, P_smyc_	This study
pSMT3-SM	Increased copy number pSMT3-S (oriM ΔA_364_)	This study
pUC57 tdTomato	codon-optimised tdTomato in pUC57	Jeff Cirillo
pASTA	codon-optimised tdTomato in pGEM-EasyT	This study
pASTA1	codon-optimised tdTomato in pSMT3	This study
pASTA2	codon-optimised tdTomato in pSMT3-M	This study
pASTA3	codon-optimised tdTomato in pSMT3-S	This study
pUC57 Turbo-365	codon-optimised Turbo-635 in pUC57	Jeff Cirillo
pCHARGE	codon-optimised Turbo-635 in pGEM Easy-T	This study
pCHARGE1	codon-optimised Turbo-635 in pSMT3	This study
pCHARGE2	codon-optimised Turbo-635 in pSMT3-M	This study
pCHARGE3	codon-optimised Turbo-635 in pSMT3-S	This study
pUC57 mCherry	codon-optimised mCherry in pUC57	This study
pCHERRY	codon-optimised mCherry in pGEM Easy-T	This study
pCHERRY1	codon-optimised mCherry in pSMT3	This study
pCHERRY2	codon-optimised mCherry in pSMT3-M	This study
pCHERRY3	codon-optimised mCherry in pSMT3-S	This study
pCHERRY4	codon-optimised mCherry in pSMT3-SM	This study
pUC57 mPlum	codon-optimised mPlum in pUC57	This study
pDUFF	codon-optimised mPlum in pGEM Easy-T	This study
pDUFF1	codon-optimised mPlum in pSMT3	This study
pDUFF2	codon-optimised mPlum in pSMT3-M	This study
pDUFF3	codon-optimised mPlum in pSMT3-S	This study
pKITTEN	codon-optimised mKat in pGEM Easy-T	This study
pKITTEN1	codon-optimised mKat in pSMT3	This study
pKITTEN2	codon-optimised mKat in pSMT3-M	This study
pKITTEN3	codon-optimised mKat in pSMT3-S	This study
pSM128	Promoter probe shuttle vector, promoterless *lacZ*, Sm^R^	[56]
pLIPPY3	*P_mbtB_* in pSM128, SmR	This study
pHIC-6	*P_mbtB_* –mCherry in replicating vector, oriM, HygR	This study

For the low iron inducible reporter, the upstream promoter region of *mbtB* was amplified from *M. tuberculosis* genomic DNA using Lippy3F (GAG TAC TGG TAC AAA GCC ACC CAG) and Lippy3R (GAG TAC TCG GAC ACC GAG CAA CTC T) and cloned into pSM128 upstream of the *lacZ* reporter [56] as a *ScaI* fragment to generate pLippy3. To generate the mCherry version, the upstream promoter region of *mbtB* was amplified using primers pHIC-6 F (CCC TCT AGA GCC GTT TGG CAG CTG GAG CAG) and pHIC-6 R (CCC GGA TCC GCC GTG CTC GGA CAT TCG C) and cloned as *Bam*HI/*Xba*I fragment into pCHERRY1, replacing P_hsp60_, to generate pHIC-6.

### Electroporation of *M. tuberculosis*

Electrocompetent *M. smegmatis*, *M. marinum* and *M. tuberculosis* cells were prepared as described previously [57], electroporated with 1 µg plasmid DNA and transformants selected with hygromycin and kanamycin where appropriate.

### Quantification of fluorescence and beta-galactosidase in liquid cultures

*E. coli*, *M. smegmatis*, *M. marinum* and *M. tuberculosis* strains were grown to stationary phase, harvested, washed twice in sterile 10 mM Tris-Cl pH 8.0 and resuspended in Middlebrook 7H9 medium plus 10% v/v OADC supplement at OD_580_ of 0.25, 0.10, 0.05 and 0.01 in 12×100 mm glass culture tubes. Fluorescence was measured on a Shimadzu RF-1501 spectrofluorimeter (Shimadzu) with a detection range of 0–1015 relative fluorescent units at the specific emission and excitation wavelengths for each fluorescent reporter; mCherry 587/610 nm, mKat 588/635 nm, mPlum 590/649 nm, tdTomato 554/581 nm and Turbo-635 588/635 nm.

### Hypoxic cultures

*M. smegmatis* strains were grown in a modified version of the Wayne model [54]. Briefly, *M. smegmatis* was grown to an OD_580_ of 0.4 in DTA medium (Becton Dickinson). For hypoxic cultures, 12×100 mm glass culture tubes containing 5 ml DTA were inoculated to an OD_580_ of 0.004 and incubated at 37°C with 6mm magnetic stirrer bars stirring at 120 rpm. For aerobic cultures 12×100mm glass cultures tubes containing 3 ml DTA were inoculated to an OD_580_ of 0.004 and incubated as above. Methylene blue (0.1%) (Sigma) was added to one culture per strain as an indicator of oxygen depletion. Fluorescence was read directly in tubes. Beta-galactosidase activity was performed as described [3].

### Macrophage infection assays

Macrophages were prepared and infected with *M. tuberculosis* as described [58]. Murine J774 monocyte macrophages [59] were infected at an MOI of 1∶1. Bacteria were harvested periodically post infection and the CFUs determined.
